# Berry-Hannay relation in nonlinear optomechanics

**DOI:** 10.1038/s41598-020-59081-5

**Published:** 2020-02-10

**Authors:** Ludovico Latmiral, Federico Armata

**Affiliations:** QOLS, Blackett Laboratory, Imperial College London, London, SW7 2AZ United Kingdom

**Keywords:** Physics, Optical physics, Quantum physics

## Abstract

We address the quantum-classical comparison of phase measurements in optomechanics in the general framework of Berry phases for composite systems. While the relation between Berry phase and Hannay angle has been proven for a large set of quadratic Hamiltonians, such correspondence has not been shown so far in the case of non-linear interactions (*e*.*g*. when three or more operators are involved). Remarkably, considering the full optomechanical interaction we recover the aforementioned mathematical relation with the Hannay angle obtained from classical equations of motion. Our results link at a fundamental level previous proposals to measure decoherence, such as the one expressed by Marshall *et al*., with the no-go theorem shown by Armata *et al*., which provides boundaries to understand the quantum-to-classical transition in optomechanics.

## Introduction

The phase acquired by a quantum state alongside its evolution is an important carrier of information and exhibits non-trivial behavior^1,2^ which has been exploited in various contexts where light interacts with matter (see ref. ^3^ and references therein) and it has been considered as a candidate for quantum gate operations in quantum information processing^4–11^. Within this framework, cavity optomechanics has revealed a promising platform to test foundations of quantum mechanics such as gravity induced deformations in commutation relations^12,13^ as well as to explore the boundary between quantum and classical mechanics and decoherence phenomena^14–20^. Thanks to substantial advances in materials science and fabrication of nano elements, experiments in this area are proliferating: miniaturization of mechanical elements has considerably increased in the last years (the mass of these mechanical objects ranges from 10^−6^ to 10^−22^ Kg)^21–23^, and has combined to the possibility of cooling the system to temperatures of the order of a few millidegres Kelvin (almost to the ground state of motion).

This promising experimental environment has induced theoretical physicists to propose experiments to witness quantum superpositions of macroscopic states. The seminal paper by Marshall *et al*.^18^ was the first in this direction. Resorting to a Michelson interferometer, the proposal consists in observing the interference pattern (*i*.*e*. the visibility) between two light fields, where one interacts with a movable mirror. The idea is to create and detect correlations between a field and a macroscopic object, and to study the creation and decoherence of superposition states through visibility loss and revival. Besides, visibility recovery has always been of fundamental importance in physics, being explained by the *quantum recurrence theorem* which generalizes the Poincaré lemma to quantum states^24–26^. All these considerations have paved the way for many fundamental studies on quantum mechanics itself and its interface with gravity as well as with classical physics^27–31^. Following this route, an intriguing problem was to clarify what could be predicted on the interaction of light fields and macroscopic objects by using only classical mechanics. Armata *et al*.^32^ addressed this problem looking at the quantum-classical correspondence of the phase acquired by an optical field after its interaction with a movable mirror in the same optomechanical setup studied in ref. ^18^. Resorting to completely different models for the classical and the quantum picture, they proved that, under common and widely adopted experimental conditions (*e*.*g*. small coupling and mechanical thermal state), the observable phase shifts of the light field coincide. This result has allowed them to challenge the visibility loss and revival as signatures respectively of quantum superposition and decoupling, and consequently as probes of any decoherence process. Instead, they have inferred that correlations arise because of (classical) statistical uncertainty on the initial state of the system and lead to effects that are qualitatively identical and quantitatively larger than those theoretically predicted for a single-photon source^18^. Conversely, they were able to isolate genuine quantum features of the interaction that appear on the phase and the visibility, which might be probed in future optomechanical experiments, even in the weak coupling limit. Nonetheless, the preparation and assessment of a truly quantum mechanical state would be an essential prerequisite for probing decoherence models or to study the interface of gravity with quantum mechanics.

Here, we aim at investigating at a fundamental level the reason behind the results in ref. ^32^, *i*.*e*. the aforementioned equality of the classical and quantum pictures and the subsequent classical revival of the visibility. Not only we will provide a deeper understanding of the phenomena, but we will also frame our findings in the much broader context of Berry phase and Hannay angle. We will show how the cyclic nature of the dynamics plays a key role in the evolution, being an essential ingredient to the quantum-classical correspondence. Our results could be easily generalised to other systems which share the same non-linear interaction^33^.

## Results

### Description of the model

**Figure 1:**
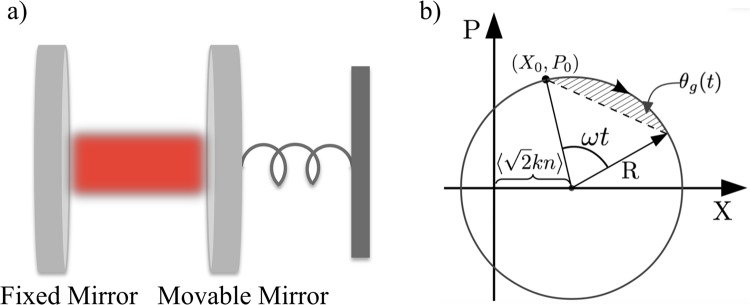
(**a**) Fabry-Perot cavity of length *L* with one fixed mirror and a movable ending mirror described as a quantum harmonic oscillator of frequency $\omega$ and mass *m*. (**b**) Representation of the mirror dynamics $(\langle X(t)\rangle ,\langle P(t)\rangle )$ in the mechanical phase space with ${X}_{0}=\sqrt{2}{\gamma }_{R}$, ${P}_{0}=\sqrt{2}{\gamma }_{I}$ and ${{\rm{R}}}^{2}=2[|\gamma {|}^{2}+{k}^{2}({N}_{p}^{2}+{N}_{p})-2{\gamma }_{R}k{N}_{p}]$ (see Methods section on the mechanical trajectory for further details).

The archetypical optomechanical system consists in a single optical field of frequency ${\omega }_{f}$ coupled to a quantum mechanical oscillator of mass $m$ and frequency $\omega$ through the radiation pressure inside an optical cavity (see Fig. 1a). The effective Hamiltonian that describes the interaction reads^15^

1 $$H=\hslash {\omega }_{f}{a}^{\dagger }a+\hslash \omega {b}^{\dagger }b-\hslash {g}_{0}{a}^{\dagger }a({b}^{\dagger }+b),$$

where $a$ (${a}^{\dagger }$) and $b$ (${b}^{\dagger }$) are the annihilation (creation) operators of the field and the mirror respectively. Equation (1) describes the so-called continuous regime, where a light pulse, after being generated and trapped in the optical cavity, will remain inside the cavity for a time of the order of the period of the mechanical oscillator. More precisely, we assume to operate in the so called *good cavity* regime, where optical damping and photon leakage from the cavity are negligible over an entire mechanical period and light and mirror continuously interact until the field escapes the cavity: this regime is defined by the condition $k\ll \omega$. In a frame rotating with the field Eq. (1) translates to the unitary evolution operator^17^

2 $$U(t)={e}^{i{k}^{2}{n}^{2}(\omega t-\sin \omega t)}{e}^{kn(\eta {b}^{\dagger }-{\eta }^{\ast }b)}{e}^{-i{b}^{\dagger }b\omega t},$$

with $n={a}^{\dagger }a$ the number operator of the field, $\eta =(1-{e}^{-i\omega t})$, $k={g}_{0}$/$\omega$, ${g}_{0}={\omega }_{f}$/$L\sqrt{\hslash /(2m\omega )}$ the coupling constant and *L* the length of the cavity at equilibrium. As we are going to show, this interaction displaces the mirror in the mechanical phase space by an average amplitude of $k{N}_{p}|\eta |$, with ${N}_{p}=\langle n\rangle$ the mean photon number of the intracavity field. In fact, let us consider the system initially prepared in the separable pure state $|\Psi (0)\rangle =|\alpha {\rangle }_{f}\otimes |\gamma {\rangle }_{m}$, with $|\alpha {\rangle }_{f}$ and $|\gamma {\rangle }_{m}=|{\gamma }_{R}+i{\gamma }_{I}{\rangle }_{m}$ coherent states of light and mirror respectively. After a time *t* the system evolves to

3 $$|\Psi (t)\rangle ={e}^{-\frac{|\alpha {|}^{2}}{2}}\,\sum _{n}\,\frac{{\alpha }^{n}}{\sqrt{n}!}{e}^{i{k}^{2}{n}^{2}(\omega t-\sin \omega t)+ikn({\gamma }_{R}\sin \omega t+{\gamma }_{I}(1-\cos \omega t))}|n{\rangle }_{f}\otimes |{\Gamma }_{n}(t){\rangle }_{m},$$

where ${\Gamma }_{n}(t)=\gamma {e}^{-i\omega t}+kn(1-{e}^{-i\omega t})$ and $|{\Gamma }_{n}(t)\rangle$ is the displaced coherent state of the mirror. We plot in Fig. 1b an example of trajectory of the mechanical oscillator in its phase space (see the Methods section for the explicit calculation of the dynamics).

The state defined in Eq. (3) acquires a geometric-like phase (${\theta }_{g}(t)$) related to the area spanned in the mechanical phase space (see Fig. 1b). While at every time $t\ne T$, with $T=2\pi$/$\omega$ the mechanical period, no deterministic measurement can be performed on either the subsystems without perturbing the state of the other, after every mechanical period the global state becomes separable, independently of the initial conditions, and the phase ${\theta }_{g}(T)$ can be measured. This is a valuable virtue of geometric phase measurements, which are resilient to several sources of error, *e*.*g*. uncertainty on the initial state of the system, thermal noise or disturbance alongside the evolution. In the specific cases of refs. ^32,34,35^ this advantage has been exploited to perform high sensitive quantum metrology requiring little initial mechanical cooling and reconstructing the dynamics via light interference.

In the next section we will discuss how this connection relates to deepest and more general physical reasons connected to a wide class of cyclic motions.

### Cyclic Evolution

When a system undergoes a cyclic evolution in quantum mechanics, the initial and final state vectors may differ by a phase factor, *i*.*e*. $|\psi (T)\rangle ={e}^{i\phi }|\psi (0)\rangle$, which can have observable consequences. This extra phase $\phi$ exclusively depends on the structure of the Hilbert space ${\mathscr{H}}$ and the geometry drawn in phase space.

The importance of such phase was firstly recognised only towards the end of the last century by Michael Berry^1^ for Hamiltonians depending on a set of parameters slowly varying in time, when the adiabatic theorem^36^ ensures that the initial state remains in an instantaneous eigenstate during the variation of the parameters. The attribute of *geometric* phase derives from its dependency on the path covered in phase space. Besides, while the proper assessment of the geometric phase is relatively recent, its classical counterpart, the *holonomic angle*, was firstly introduced in 1900 by Tullio Levi Civita^37^, who formulated the concept of the *parallel transport* of vectors on a surface. This consists in translating a vector alongside a closed path *C*, always keeping it tangent to the surface Ω which contains *C*. After a closed loop the vector is rotated with respect to its original orientation by an angle whose amplitude and sign respectively depend on the geometry of the loop and on the direction along which the vector was displaced. Foucault pendulum is probably the most popular device used to provide physical evidence of this mathematical peculiarity that has a more general interpretation: in non-holonomic systems path integrals depend on the whole state evolution path in Hilbert space.

The first rigorous analysis of these properties is due to John Hannay^38^, who found an algebraic relation between the quantum geometric Berry phase and the classical Hannay angle^38,39^. Thereafter, the connection between the two pictures has been proven for a wide set of quadratic Hamiltonians (*e*.*g*. containing *q*^2^, *p*^2^ or *qp*) and in a range of different contexts^40,41^. Hence, the aim of this paper is also to apply Berry and Hannay formalisms within the context of two-systems interactions, providing a complete understanding of the theoretical structure underlying the optomechanical (and the ion trap) dynamics. In particular, since the light-matter interaction is regulated by a non-linear cubic term, *i*.*e*. $g{a}^{\dagger }a({b}^{\dagger }+b)$, and leads to non-gaussian states, the retrieved quantum-classical correspondence will be of great interest.

### Berry phase

We start by looking at the system in a quantum picture to evaluate the geometrical and dynamical contributions to the quantum phase^1^. To this end, we follow the approach in^42^ where phase changes in cyclic evolutions have been considered and adiabaticity condition has been relaxed.

Given a Hilbert space ${\mathscr{H}}$ where a state vector that undergoes a closed loop acquires a phase factor $\phi$, *i*.*e*. $|\psi (T)\rangle ={e}^{i\phi (T)}|\psi (0)\rangle$, we consider the Abelian gauge transformation $\Pi (|\psi \rangle )$ = {$|\psi ^{\prime} \rangle :|\psi ^{\prime} \rangle =c|\psi \rangle$, with *c* a complex number} that projects the states on a new Hilbert space ${\mathscr{H}}^{\prime}$, $\Pi :{\mathscr{H}}\to {\mathscr{H}}^{\prime}$, where after one period the evolution is actually closed: $|\psi ^{\prime} (T)\rangle =|\psi ^{\prime} (0)\rangle$, being $|\psi (t)\rangle ={e}^{if(t)}|\psi ^{\prime} (t)\rangle$, with $f(t)-f(0)=\phi$. By suing the Schrödinger equation for $|\psi (t)\rangle$, we have

4 $$-\,\dot{f}=\frac{1}{\hslash }\langle \psi (t)|H|\psi (t)\rangle -\langle \psi ^{\prime} (t)|i\frac{\partial }{\partial t}|\psi ^{\prime} (t)\rangle .$$

Integrating the last equation over a path *C* in phase space one obtains ${\int }_{0}^{t}\,\dot{f}d\tau =\phi ={\theta }_{d}+{\theta }_{g}$ with

5 $$\begin{array}{rcl}{\theta }_{d}(t) & = & \frac{1}{\hslash }\,{\int }_{0}^{t}\,d\tau \langle \psi (\tau )|H|\psi (\tau )\rangle \,{\rm{and}}\\ {\theta }_{g}(t) & = & -\,i\,{\int }_{0}^{t}\,d\tau \langle \psi ^{\prime} (t)|\frac{\partial }{\partial \tau }|\psi ^{\prime} (\tau )\rangle ,\end{array}$$

where ${\theta }_{d}(t)$ and ${\theta }_{g}(t)$ are defined as the dynamic and geometric (or Berry) phase respectively. Relevantly, the pulsed Hamiltonian *H* is time independent and *θ*_*d*_ can be calculated easily: substituting from Eq. (3) the state $|\Psi (t)\rangle$ in Eq. (5) we get for a generic time *t*

6 $${\theta }_{d}(t)=(2{\gamma }_{R}k{N}_{p}-|\gamma {|}^{2})\omega t.$$

The calculation for ${\theta }_{g}$ is a bit more laborious since to compute the argument of the integral (also known as *Berry connection*) one needs both to solve the dynamics of the system and the map $\Pi$. In this direction, it is helpful for our purpose to reconsider the evolution operator in Eq. (2) and observe that it is diagonal in the cavity Fock state basis. Thanks to this property, the state in Eq. (3), offers a decomposition that automatically satisfies

7 $$|\Psi (t)\rangle =\mathop{\sum }\limits_{n=0}^{\infty }\,{e}^{i{f}_{n}(t)}|\Psi ^{\prime} (t){\rangle }_{n},$$

with $|\Psi ^{\prime} (t){\rangle }_{n}=|n{\rangle }_{f}\otimes |{\Gamma }_{n}(t){\rangle }_{m}$. Substituting this decomposition in Eq. (5) and remembering that the derivative of a coherent state $\frac{\partial }{\partial t}|\Gamma \rangle$ gives $\langle \Gamma |\frac{\partial }{\partial t}|\Gamma \rangle =-\,\frac{\partial }{\partial t}\frac{|\Gamma {|}^{2}}{2}+{\Gamma }^{\ast }\frac{\partial \Gamma }{\partial t}$, we obtain the *Berry phase*

8 $$\begin{array}{rcl}{\theta }_{g}(t) & = & (|\gamma {|}^{2}-2{\gamma }_{R}k{N}_{p})\omega t+k{N}_{p}[{\gamma }_{I}(1-\,\cos \,\omega t)+{\gamma }_{R}\,\sin \,\omega t]\\  &  & +\,{k}^{2}({N}_{p}^{2}+{N}_{p})\,(\omega t-\,\sin \,\omega t).\end{array}$$

**Figure 2:**
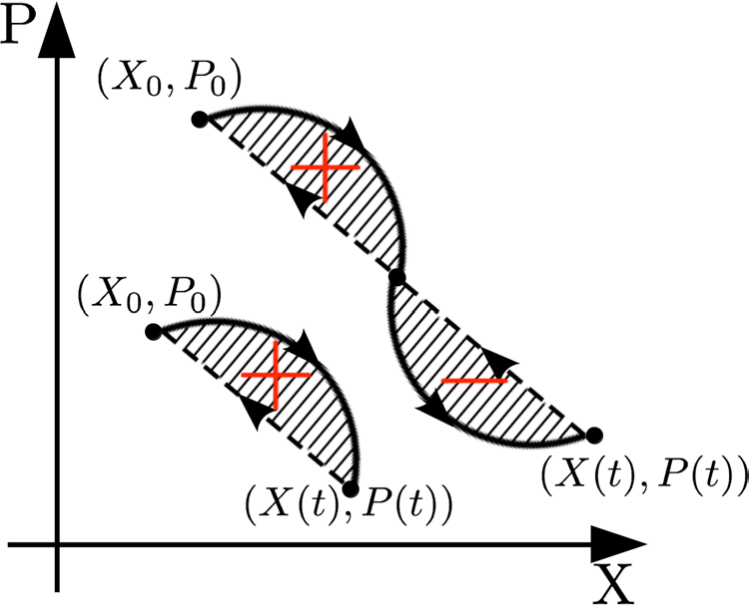
Representation of the geometric phase in phase space. The trajectory (of the mirror) is depicted with a continuum line and the geometric phase corresponds to the shaded region delimited by the trajectory and by the dashed line that connects $(X(t),P(t))$ to $({X}_{0},{P}_{0})$. As a convention, areas enclosed clockwise are positive, those travelled anti-clockwise negative.

Even though it is not obvious at first sight, ${\theta }_{g}(t)$ corresponds to the dashed area in Fig. 1b spanned in phase space by the line segment that joins the instantaneous position to the starting point of the dynamics. This expains the reason of the name “geometric phase” for the quantity expressed in Eq. (8). The intuitive idea behind a geometric phase defined at all times $t\in [0,T]$ and not only at each period *T*, *i*.*e*. for open paths in phase space, is that one can close the loop by judiciously choosing a “geodesic”, *i*.*e*. the straightest path according to the Study-Fubini metric^2^ (see Fig. 2 for a graphical representation).

Moreover, our optomechanical system enjoys another important property: even if each component ${\theta }_{g}(t)$, ${\theta }_{d}(t)$ depends on the initial conditions, after a mechanical period $T=2\pi$/$\omega$ their sum $\phi (T)={\theta }_{g}(T)+{\theta }_{d}(T)=2\pi {k}^{2}{N}_{p}^{2}$ solely depends on the geometry of the evolution in space. More specifically, it is instructive to consider the limit of small coupling $k\ll 1$, which corresponds to the state of the art in current optomechanical experiments^22^. In this limit, indeed, the initial coherent state of the light $|\alpha {\rangle }_{f}$ (and the state vector $|\Psi (t)\rangle$ in Eq. (3)) is approximately an eigenstate of the Hamiltonian: the phase $\phi (t)$ thus coincides with the global phase we could attribute to the quantum superposition state of the system: $|\Psi (t)\rangle ={e}^{i\phi (t)}|\Psi (0)\rangle$.

### Hannay angle

Non-dissipative models are usually associated with a conserved quantity, namely the total energy (or the number of photons in our case). Within this class of models, periodic motions deserve special attention, as many peculiarities can be sorted out by simply applying a canonical transformation of second type. To this end, it is convenient to switch from the Hamiltonian classical variables $(q,p)$ to the so-called *action*-*angle* coordinate system $(\phi ,I)$, which is characterised by a conserved quantity *I* (usually representing the total energy), that unambiguously identifies the *orbit* in phase space, and a periodic variable $\phi$, that univocally determines the position of the system on the orbit at any given time^43^. A possible candidate as *generating* function to provide the desired transformation is

9 $$S(q,I)={\int }_{{q}_{0}}^{q}\,p(q^{\prime} ,I)dq^{\prime} ,$$

where the integral is performed along the orbit defined by *I*, *e*.*g*. with fixed and constant energy. The canonically conjugate variables can then be derived from $S(q,I)$ as $p=\frac{\partial S}{\partial q}$, which follows straightforwardly by simple substitution in Eq. (9), and $\phi =\frac{\partial S}{\partial I}$.

To further proceed with a classical interpretation of an electromagnetic field continuously interacting with a mirror in an optomechanical cavity we should introduce the related Hamiltonian. The field exerts a constant force during the whole evolution, whose strength is derived from the impulse-momentum theorem $F=\partial P/\partial t=2{E}_{0}/(cdt)={E}_{0}/L$, with *E*_0_ the field energy. The classical Hamiltonian and its solution (analogous to Eqs. (1)–(15)) will then read

10 $$\begin{array}{rcl}{H}_{c} & = & \frac{1}{2}m{\omega }^{2}{x}^{2}+\frac{1}{2m}{p}^{2}-\frac{{E}_{0}}{L}x,\,{\rm{and}}\\ x(t) & = & x(0)\,\cos \,\omega t+\frac{p(0)}{m\omega }\,\sin \,\omega t+\frac{{E}_{0}}{m{\omega }^{2}L}(1-\,\cos \,\omega t).\end{array}$$

Substituting the optomechanical parameters and bearing in mind that ${E}_{0}=\hslash {\omega }_{c}{N}_{p}$ and that the initial displaced Gaussian quantum state ${\gamma }_{m}$ corresponds to the classical boundary conditions $x(0)=\sqrt{2}{\gamma }_{R}\sqrt{\hslash /(m\omega )}$ and $p(0)=\sqrt{2}{\gamma }_{I}\sqrt{\hslash m\omega }$, we verify the correspondence with the quantum dynamics found in the Methods.

Let us then calculate the phase $\phi$ related to our case of interest, generalising the procedure rigorously addressed in refs. ^38,39^ to non-closed loops in phase space by applying the same technique we presented in the last section to artificially close the path at each time *t*.

We know that a second type generating function (as the one in Eq. (9)) transforms the Hamiltonian as $H^{\prime} =H+\partial S/\partial t$: we can thus derive the phase change rate from Hamilton equation as

11 $$\dot{\phi }=\frac{\partial H^{\prime} }{\partial I}=\frac{\partial H}{\partial I}+\frac{{\partial }^{2}\tilde{S}}{\partial t\partial I}-\frac{\partial }{\partial I}\frac{\partial }{\partial t}pdq,$$

where we have expressed $\partial S$/$\partial I$ in terms of the new angle-action variables: $d\tilde{S}(\phi ,I)=dS(q,I)+\frac{\partial S}{\partial q}dq$, with $I={E}_{0}$/${\omega }_{c}$ (corresponding to $\hslash$*N*_*p*_ in the quantum framework).

The classical phase associated with a (closed) path *C* in phase space is then obtained by integrating

12 $$\phi (t)={\int }_{0}^{t}\,\frac{\partial H}{\partial I}d\tau -\frac{\partial }{\partial I}\,{\int }_{C}\,pdq,$$

where we have exploited that ${\int }_{C}\,\frac{\partial \tilde{S}}{\partial I}=0$, *i*.*e*. it is the average of the derivative of a periodic function, and we have considered a generic time *t*, as long as the loop is artificially closed with a geodesic arc as depicted in Fig. 2.

We notice that Eq. (12) consists of two parts that are related with the dynamical and geometric phase state components introduced in Eq. (5) which read respectively

13 $$\begin{array}{rcl}{\phi }_{d}(t) & = & {\int }_{0}^{t}\,\frac{\partial H}{\partial I}d\tau =\frac{{\omega }_{c}x(0)t}{L},\,{\rm{and}}\\ {\phi }_{g}(t) & = & -\,\frac{\partial }{\partial I}\,{\int }_{C}\,pdq=-\,\frac{{\omega }_{c}x(0)t}{L}+\frac{{\omega }_{c}}{{\omega }^{3}m{L}^{2}}{E}_{0}(\omega t-\,\sin \,\omega t)\\  &  & +\,\frac{{\omega }_{c}}{2L\omega }[x(0)\,\sin \,\omega t+\frac{p(0)}{m\omega }(1-\,\cos \,\omega t)].\end{array}$$

${\phi }_{g}(t)$ is usually referred to as Hannay angle, named after the physicist John H. Hannay, who first identified this quantity in adiabatic classical systems as the derivative of the Berry phase with respect to the action variable *I*

14 $${\phi }_{g}(t)=\frac{d{\theta }_{g}(t)}{dI}.$$

Hannay equation can be explained as an application of Stokes theorem: since we are considering a closed loop (either artifcially if $t\in [0,T)$ or not if $t=T$), ${\phi }_{g}(t)$, *i*.*e*. the derivative of the area enclosed by the trajectory, should also be equal to the path integral over the position of the mechanical oscillator of the transferred momentum from the field to the mirror *p*. As we show in the Methods section referring to the analysis presented in ref. ^32^, this result can be obtained independently, resorting to basic rules of classical optics. Stokes theorem thus helps us to generalize the quantum-classical correspondence and the Berry-Hannay relation, which we have shown for a non-quadratic Hamiltonian: replacing the quantum parameters with their classical average expectation values in Eq. (8), we see that Eq. (13) solves Eq. (14). Noticeably, ${\phi }_{g}(t)$ is the only measurable quantity, while ${\varphi }_{c}(t)={\phi }_{g}(t)+{\phi }_{d}(t)$ (also computed in Methods) is the most relevant one since it is periodically independent of the initial conditions.

## Discussion

In this paper we addressed the study of Berry phase and Hannay angle in optomechanics. We thus generalized the quantum-classical correspondence that was discussed in ref. ^32^, where the readout of the dynamics was proven to be the same in the two pictures, recurring to completely different approaches. That result has severely questioned the quantumness of optomechanical systems, and hence their employment to test quantum peculiarities (*e*.*g*. deviations in commutation relations, creation of macroscopic quantum states, or probing decoherence). Conversely, we have now attributed such quantum-classical correspondence to the wider framework of the Berry-Hannay relation, as we successfully verified that also for the cubic non-linear optomechanical interaction the quantal geometric phase causes the state to be displaced along the classical trajectory by an amount equal to the classical geometric phase (Hannay angle)^44^. Interestingly, the results obtained in this paper could be easily extended to the other regime that was investigated in ref. ^32^, *i*.*e*. the so called pulsed or bad cavity regime. From a fundamental perspective, contextualizing optomechanics within the more general framework of non-abelian transformations in phase space paves new ways for future investigations on the quantum-classical transition in light of the Berry-Hannay relation^45^.

On the other hand, from an experimental perspective, the resilience of geometric phases to various sources of error, *e*.*g*. uncertainty on the initial state of the system and thermal noise, makes them an ideal candidate to perform high-fidelity phase-gate operations^46^, which are pivotal to new applications in quantum computing, and quantum metrology^47,48^.

## Methods

### Mechanical trajectory

We provide hereafter the calculation of the expected values of the mechanical oscillator in phase space obtained by averaging the mechanical position $X=({b}^{\dagger }+b)$/$\sqrt{2}$ and momentum $P=i({b}^{\dagger }-b)$/$\sqrt{2}$ on the initial state $|\gamma {\rangle }_{m}$ using the unitary operator in Eq. (2). We obtain

15 $$\begin{array}{rcl}\langle X(t)\rangle /\sqrt{2} & = & {\gamma }_{r}\,\cos \,\omega t+{\gamma }_{i}\,\sin \,\omega t+k{N}_{p}(1-\,\cos \,\omega t)\,,\\ \langle P(t)\rangle /\sqrt{2} & = & {\gamma }_{i}\,\cos \,\omega t-{\gamma }_{r}\,\sin \,\omega t+k{N}_{p}\,\sin \,\omega t\,.\end{array}$$

### Phase shifts with classical continuous interaction

From a classical perspective, the Doppler effect causes a phase shift when a field is reflected by movable mirror which is proportional to the product of the field wavevector k_*f*_ and the mirror position: ${\varphi }_{c}(x)=2{{\rm{k}}}_{f}\,\int \,vdt=2{{\rm{k}}}_{f}\,\oint \,dx$, where the path-integral extends over all the positions occupied by the reflective mirror during the interaction.

The phase acquired during a continuous evolution could then be calculated as

16 $$\begin{array}{rcl}{\varphi }_{c}(t) & = & 2\frac{{{\rm{k}}}_{f}}{d\tilde{\tau }}\,{\int }_{0}^{t}\,x(\tau )d\tau =\frac{{\omega }_{c}}{L\omega }[x(0)\,\sin \,\omega t+\frac{p(0)}{m\omega }(1-\,\cos \,\omega t)]\\  &  & +\,\frac{{\omega }_{c}}{{\omega }^{3}m{L}^{2}}{E}_{0}(\omega t-\,\sin \,\omega t),\end{array}$$

with $d\tilde{\tau }=2L/c$ the round trip time of light inside the cavity. If we were to close the loop with a geodesic line we would add a contribution equal to $-\,\frac{{\omega }_{c}x(0)t}{L}$, thus obtaining exactly the same result as in Eq. (13).

Interestingly, as suggested in ref. ^32^, this analogy is not limited to the continuous - good cavity regime, but is preserved also in a pulsed - bad cavity regime, when an intense and short light pulse enters the cavity and quickly escapes from it.

### Phase measurements in optomechanics

Hereafter we provide details on how phase measurements can be performed in an optomechanical cavity, retrieving information on the quantum state of the system. We will discuss homodyne detection which corresponds to projecting on the quadrature operator ${X}_{\phi }=(1/\sqrt{2})[a{e}^{-i\phi }+{a}^{\dagger }{e}^{i\phi }]$, where *a* is the optical field operator that exits the cavity. The first step to compute the expectation value of ${X}_{\phi }$ is therefore to evaluate the mean value of the optical field $\langle a\rangle ={\rm{Tr}}\,[a{\rho }_{f}]$, where ${\rho }_{f}$ is the reduced density matrix of the field. Hence, from the state in Eq. (3), where the mirror is initially in a coherent state $|\gamma \rangle$, we compute the total density operator of the system

17 $$\begin{array}{rcl}\rho (t) & = & {e}^{-|\alpha {|}^{2}}\,\sum _{m,n}\,\frac{{\alpha }^{n}{\alpha }^{\ast m}}{\sqrt{n!m!}}{e}^{i{k}^{2}({n}^{2}-{m}^{2})(\omega t-\sin \omega t)}\\  &  & \times \,{e}^{ik(n-m)({\gamma }_{r}\sin \omega t+{\gamma }_{i}(1-\cos \omega t))}|n{\rangle }_{f}{\langle m|\otimes |{\Gamma }_{n}(t)\rangle }_{m}\langle {\Gamma }_{m}^{\ast }(t)|.\end{array}$$

This expression is suitable to retrieve the reduced density matrix of field when the mirror is initially in a thermal state. This in fact corresponds to a statistical mixture of coherent states defined by ${\rho }_{m}={(\pi \bar{n})}^{-1}\,\int \,{d}^{2}\gamma {e}^{-|\gamma {|}^{2}/\bar{n}}|\gamma {\rangle }_{m}\langle \gamma |$, where $\bar{n}=1/({e}^{\hslash \omega /({k}_{b}T)}-1)$ is the average thermal occupation number. By computing such a weighted average with the expression in Eq. (17) and tracing out over the mechanical degrees of freedom we obtain ${\rho }_{f}$

18 $${\rho }_{f}(t)={e}^{-|\alpha {|}^{2}}\,\sum _{m,n}\,\frac{{\alpha }^{n}{\alpha }^{\ast m}}{\sqrt{n!m!}}{e}^{i{k}^{2}({n}^{2}-{m}^{2})(\omega t-\sin \omega t)-{k}^{2}{(n-m)}^{2}(1-\cos \omega t)(2\bar{n}+1)}|n{\rangle }_{f}\langle m|.$$

This allows us to calculate the average value of the fled operator at time *t*

19 $$\langle a\rangle =\alpha {e}^{-{k}^{2}(1-\cos \omega t)(2\bar{n}+1)}{e}^{-{N}_{p}\{1-\cos [2{k}^{2}(\omega t-\sin \omega t)]\}}{e}^{i{k}^{2}(\omega t-\sin \omega t)+i{N}_{p}\sin [2{k}^{2}(\omega t-\sin \omega t)]},$$

from which we infer the phase acquired by the optical field $\phi (t)={k}^{2}(\omega t-\,\sin \,\omega t)+{N}_{p}\,\sin \,[2{k}^{2}(\omega t-\,\sin \,\omega t)]$.
